# Mapping the Evidence on Compassion Skills in Applied Behavior Analysis: Protocol for Scoping Review

**DOI:** 10.2196/66399

**Published:** 2025-04-24

**Authors:** Daniele Nunes Longhi Aleixo, Daiton Junior Martins de Souza, Stela Regina Pedroso Vilela Torres de Carvalho, Marcia Regina Furlani, Cíntia Canato Martins, Emerson Roberto dos Santos, João Daniel de Souza Menezes, Matheus Querino da Silva, Sônia Maria Maciel Lopes, Marcos Sanches Rodrigues, Natalia Almeida de Arnaldo Silva Rodriguez Castro, Helena Landim Gonçalves Cristóvão, Josimerci Ittavo Lamana Faria, Vânia Maria Sabadoto Brienze, Alba Regina de Abreu Lima, Patrícia da Silva Fucuta, Denise Cristina Móz Vaz Oliani, Neide Aparecida Micelli Domingos, Maria Cristina Oliveira Santos Miyazaki, Gerardo Maria de Araújo Filho, Júlio César André

**Affiliations:** 1 Faculty of Medicine of São José do Rio Preto (FAMERP) Center for Studies and Development of Health Education (CEDES) São José do Rio Preto Brazil; 2 Neurodevelopmental Diagnosis and Intervention Center (CEDIN) Curitiba Brazil; 3 University of Santo Amaro São Paulo Brazil; 4 Faculty of Medicine of São José do Rio Preto (FAMERP) Ceres School of Medicine (FACERES) São José do Rio Preto Brazil

**Keywords:** compassion, applied behavior analysis, skills, protocol, scoping reviews, knowledge gaps, training program, client-centered approach, holistic approach

## Abstract

**Background:**

Applied behavior analysis (ABA) is a scientific approach that applies principles of learning and motivation to assess, design, implement, and evaluate social and environmental modifications to produce meaningful changes in human behavior. It has been widely used in various settings, particularly in the treatment of individuals with autism spectrum disorders and other developmental disabilities. Recently, compassion has emerged as a topic of growing scientific interest within ABA. To improve socially relevant behaviors, it is essential to explore how behavior analysts can provide maximum support to clients and promote significant changes through compassionate care. Although compassion skills have been studied by ABA researchers, the literature still presents gaps in understanding how these skills can be effectively integrated into ABA practice.

**Objective:**

This study aimed to map, identify, and provide data available in the existing literature on compassion skills and applied behavior analysis.

**Methods:**

This scoping review will follow the methodological framework of Arksey and O’Malley with previously proposed refinements. The search strategy will use combinations of descriptors and their synonyms according to the Health Sciences Descriptors and MeSH (Medical Subject Headings) terms, using the PCC (population, concept, and context) mnemonic, combined with the Boolean operators AND Mesh OR. The electronic databases to be searched include Embase, Index Psicologia, Lilacs, PubMed, Scopus, and Web of Science. Studies published between 2020 and 2024 in English, Portuguese, and Spanish will be included. Two independent reviewers will screen titles, abstracts, and full texts, with a third reviewer resolving any disagreements.

**Results:**

As this is a protocol, results are pending. The review will synthesize definitions of compassion in ABA, map compassionate skills, analyze existing interventions, and identify outcomes associated with compassionate ABA practice.

**Conclusions:**

This scoping review is expected to contribute to the evolution of ABA toward a more compassionate and holistic approach, potentially leading to improved outcomes for clients and practitioners. The findings may inform the development of compassion training programs and influence care policies in ABA.

**Trial Registration:**

OSF Registries 10.17605/OSF.IO/F3A6H; https://osf.io/f3a6h

**International Registered Report Identifier (IRRID):**

PRR1-10.2196/66399

## Introduction

### Background

Applied behavior analysis (ABA) is an approach widely recognized as effective in improving the quality of life of people with autism spectrum disorder (ASD) [1]. This approach is based on the principles of behaviorism and aims to modify behaviors through systematic and evidence-based interventions [2]. With continuous advancements in this field, recent studies have demonstrated ABA’s efficacy not only for individuals with ASD but also for a variety of other neurodevelopmental conditions [3] as the field progresses and scientific interest in humanized interventions grows, the concept of “compassion” has become a topic of discussion, with new skills, such as compassionate care, becoming central to therapeutic process [4]. The incorporation of compassionate practices in ABA has been associated with improved therapeutic outcomes and greater satisfaction among clients and their families [5]. Recent research has further emphasized the importance of concrete compassionate actions in building compassionate experiences within health care settings, including ABA practice [6].

Compassion can be defined as a combination of empathy, care, and willingness to help others, alleviating their suffering [7]. In the context of health care, compassion is considered an essential component for the quality of care and patient satisfaction [8]. To improve the quality of health care, it is essential to focus on a compassionate and client-centered approach, aiming for better outcomes.

However, despite the growing recognition of the importance of compassion, studies have pointed out complaints from those who receive ABA services about the attitudes and behaviors of these professionals, indicating the need to develop strategies that promote more humanized care [3]. These complaints include lack of empathy, inadequate communication, and overly directive approaches by behavior analysts [9].

Although ABA has brought several significant contributions to the treatment of ASD, there is a lack of aspects that encompass the conceptual and operational definition of compassion from a behavioral perspective [10]. Recently, researchers have endeavored to bridge this gap, proposing conceptual models of compassion aligned with the principles of behavior analysis [10]. Most studies on compassion have focused on specific populations, leaving a gap in knowledge about how to develop and apply compassionate skills in different contexts [5]. Furthermore, the integration of compassionate skills in the training and practice of behavior analysts has gained increasing attention in recent literature [9].

These gaps indicate the need for a comprehensive review to map research data on the compassionate skills of the behavior analyst. The importance of such a review is underscored by the growing recognition of compassion as a crucial component in delivering high-quality health care [8]. Unlike other reviews, a scoping review is particularly suitable, as it will allow the identification of critical resources of the ABA professional’s compassion skills, analyzing knowledge gaps and providing a comprehensive view of compassion skills in this area of concentration. This type of review has proven effective in mapping evidence in emerging and multidisciplinary fields, such as the integration of compassion in ABA [2]. Furthermore, the scoping review will enable the synthesis of evidence from different types of studies, such as clinical trials, observational studies, and qualitative research [11].

Recent developments in the field have also highlighted the need for standardized measures of compassion in behavioral interventions. Marchese and Weiss [1] have made significant strides in this direction by developing the Parent Partnership Questionnaire, a tool designed to assess compassionate care in ABA settings. This advancement provides a foundation for more rigorous evaluation of compassionate practices and their impact on treatment outcomes.

In addition, the evolving landscape of ABA practice has seen an increased emphasis on cultural responsiveness and empathy as integral components of compassionate care. This shift reflects a growing awareness of the diverse needs of clients and the importance of tailoring interventions to individual and cultural contexts [4]. Such developments underscore the necessity for a comprehensive review that can synthesize these emerging trends and provide direction for future research and practice in the field.

### Objective

The primary objective of this scoping review is to map and synthesize the available evidence on the compassionate skills of behavior analysts, identifying knowledge gaps and providing directions for future research and clinical practices.

The specific objectives are:

- To identify the definitions and conceptualizations of compassion in the context of ABA.
- To map the specific compassionate skills reported in the ABA literature.
- To analyze existing interventions or training programs for developing compassionate skills in behavior analysts.
- To examine the reported outcomes associated with compassionate practice in ABA.
- To identify barriers and facilitators for implementing compassionate practices in ABA.

It is expected that the results of this review will contribute to the improvement of ABA professionals’ training and performance, promoting more humanized care focused on clients’ needs.

## Methods

### Study Design

This study is a scoping review protocol. This review model was selected because it is a specific area of interest with little coverage of the topic in the current literature. The review will follow the methodological framework of Arksey and O’Malley [12], with refinements proposed by Levac et al [13] and Peters et al [11]. The results will be presented using the PRISMA-ScR (Preferred Reporting Item for Systematic Review and Meta Analyses extension for Scoping Reviews) guidelines [14] to ensure transparency and quality of the review process.

### Research Question

To answer the research question, we considered the target population and the concept to refine the formulation of the PCC (population, concept, and context) acronym. Therefore, the scoping review question was defined as: “What has been widely published in the scientific literature about the compassion skills of the Behavior Analyst?”

The acronym encompasses the components of Population, Concept, and Context as follows: the Population refers to applied behavior analysts, the Concept revolves around compassion, and the Context is related to professional skills and competencies within ABA practice.

In this scoping review, the “context” element of our PCC framework refers to the professional skills and competencies within ABA practice. This encompasses the specific abilities, techniques, and knowledge that behavior analysts use in their professional roles. By focusing on this context, we aim to explore how compassion is integrated into the skillset of ABA practitioners, including their interpersonal skills, therapeutic techniques, and overall approach to client care. This context allows us to examine compassion not as an isolated concept, but as an integral part of the professional competencies required in the field of ABA.

### Inclusion and Exclusion Criteria

The inclusion criteria will comprise peer-reviewed studies in Portuguese, English, and Spanish, with any research design that answers the guiding question. The studies will need to present complete abstracts in the databases available accessed via internet web, free of charge, in full, published between 2020 and 2024 (Textbox 1), and referring to research related to the area of concentration of Applied Behavior Analysis. All databases will be consulted through the CAPES Journal Portal.

**Justification for the 2020-2024 time frame.**

**Rapid evolution of the field:** The integration of compassion into applied behavior analysis practices has experienced significant acceleration in recent years. By focusing on the last 5 years, we aim to capture the most current and relevant developments in this rapidly evolving field.

**Paradigm shift:** The period since 2020 marks a notable shift in applied behavior analysis toward more holistic, person-centered approaches, with increased emphasis on compassionate care. This time frame allows us to concentrate on literature that reflects this important paradigm shift.

**COVID-19 impact:** The global pandemic has profoundly influenced health care delivery, including applied behavior analysis services, potentially affecting the implementation and perception of compassionate care. Literature from 2020 onwards is more likely to reflect these crucial changes.

**Methodological advancements:** Recent years have seen significant improvements in research methodologies and measurement tools related to compassion in behavioral interventions. Focusing on this period ensures we capture studies using the most advanced and validated methods.

**Policy and guideline updates:** Many professional organizations have updated their guidelines and ethical standards in recent years to incorporate compassionate care principles. Literature from 2020-2024 is more likely to reflect these updated standards.

**Alignment with current practice:** By concentrating on the most recent literature, we ensure that our findings are highly relevant to current clinical practice and ongoing research initiatives.

**Manageability of data:** Given the comprehensive nature of a scoping review, limiting the time frame to 5 years helps ensure a manageable volume of data while still providing a robust representation of current knowledge.

To ensure comprehensive coverage of relevant research methodologies in ABA, we will explicitly include Single-Case Experimental Designs (SCEDs) in our inclusion criteria. SCEDs are experimental in nature and distinct from descriptive case studies. These designs are widely used in ABA research due to their ability to demonstrate functional relationships between interventions and behavior changes at the individual level. By including SCEDs, we acknowledge their significance in the field and ensure that valuable data from these rigorous experimental designs are captured in our review. Specifically, we will include studies using various SCED methodologies, such as multiple baseline designs, reversal designs, alternating treatments designs, and changing criterion designs. This inclusion will allow for a more comprehensive analysis of compassionate practices in ABA across diverse research methodologies.

While we acknowledge that some seminal works cited in our introduction predate 2020, these are primarily used to provide historical context and theoretical foundations. Our focus on 2020-2024 for the scoping review itself allows us to build upon this foundational knowledge with the most up-to-date empirical evidence and practical applications.

We believe this approach strikes an optimal balance between capturing the most current developments in the field and maintaining a manageable scope for our review. However, we remain open to expanding this time frame if our initial search reveals a paucity of relevant literature within the specified period.

Studies will be included only if they are available in full text, either through open access or free availability. Papers that provide only the abstract free of charge will not be included in this review. This decision has been made to ensure a comprehensive and in-depth analysis of the entire content of selected studies, allowing for a more robust and reliable assessment of the evidence. We acknowledge that this choice may limit the scope of the review, potentially excluding some relevant studies that are available only behind paywalls. However, we posit that the benefits of full-text analysis outweigh this potential limitation. To mitigate this constraint, we will conduct a thorough search across multiple databases and, where possible, use institutional access to maximize our coverage of relevant literature. In addition, in cases where a potentially crucial paper is not freely available, we will attempt to contact the authors directly to request access. This approach aims to balance the need for comprehensive analysis with the practical constraints of access to scientific literature.

The exclusion criteria in this study will include research published more than 5 years ago and paper that are not related to the area of concentration of ABA, as well as other items shown in Textbox 2.

**Inclusion and exclusion criteria.**

Inclusion criteria

- Quantitative studies.
- Qualitative studies.
- Mixed methods studies.
- Experimental and quasi-experimental design studies.
- Randomized controlled studies.
- Non-randomized controlled studies.
- Systematic reviews.
- Meta-analyses
- Meta-syntheses.
- Books and guidelines published in indexed sources.

Exclusion criteria

- Editorials.
- Expert opinions.
- Narrative reviews.
- Studies that do not contemplate applied behavior analysis.

### Search Strategy and Sources of Evidence

The search for published papers will be processed in the following electronic databases: Embase, Index Psicologia, Lilacs, PubMed, Scopus, and Web of Science. These specific databases were selected because they have been used in previous review studies. Based on their comprehensive coverage of behavioral and psychological research, as well as their use in previous systematic reviews in the field of ABA and compassion in health care. Specifically:

- Embase and PubMed were chosen for their extensive coverage of biomedical literature, including behavioral interventions [15].
- Index Psicologia and Lilacs were included to ensure comprehensive coverage of Latin American and Caribbean health science literature, which is particularly relevant given our inclusion of Portuguese and Spanish language studies [16].
- Scopus and Web of Science were selected for their broad, multidisciplinary coverage and their inclusion of high-impact journals in psychology and behavioral sciences [17].

This combination of databases ensures a thorough and diverse search across relevant disciplines and geographical regions.

The search strategy used combinations of descriptors and their synonyms according to the health sciences descriptors and MeSH (Medical Subject Headings) terms, using the PCC mnemonic, combined with the Boolean operators AND and OR.

The research team developed and tested the search strategy on the CAPES Journal Portal with the support of a librarian specializing in the subject. The search strategy is presented in Textbox 3. Once the appropriate papers have been identified and duplicates removed, the literature analysis will begin, and if appropriate, the search strategy in the literature list will be refined accordingly. The search strategy is outlined in Table 1.

It is important to note that the term “skills” was listed as a replacement for the descriptor “Abilities” to refine the keywords established for the study and their alternative terms.

The searches were performed using descriptors in Portuguese, Spanish, and English. In Spanish, the descriptors were also used in fields such as “title words” and “abstract word” for greater coverage, but without any results.

The descriptors were inserted into the protocol, along with their respective alternative terms. For greater results, in some databases, only the main descriptor was inserted for the search.

**Selection of keywords for search strategy.**

“Habilidades” OR **“Aptidão”** OR “Habilidade Pessoal” OR “Habilidades para a Vida” OR “Talento” OR **“Aptitudes”** OR “Talent” OR “Talents” OR “Ability” OR “Abilities” OR **“Aptitud” AND “Empatia”** OR “Benevolência” OR “Compaixão” OR “Condescendência” OR “Consideração” OR “Deferência” OR “Sentimento de Complacência” OR **“Empathy”** OR “Caring” OR “Compassion” OR **“Empatía”** AND **“Análise do Comportamento** Aplicada” OR **“Applied Behavior Analysis”** OR **“Análisis Aplicado de la Conducta”**

**Table 1:** Search strategy.

Term	Synonyms in Portuguese (descriptors)	Synonyms in English (MeSH^a^)	Synonyms in Spanish (descriptors)
Term 1	“Habilidades” OR “Aptidão” OR “Habilidade Pessoal” OR “Habilidades para a Vida” OR “Talento”	“Aptitudes”* OR “Talent”* OR “Talents”* OR “Ability”* OR “Abilities”*	“Aptitud”
Term 2	“Empatia” OR “Benevolência” OR “Compaixão” OR “Condescedência” OR “Consideração” OR “Deferência” OR “Sentimento de Complacência”	“Empathy” OR “Caring” OR “Compassion”	“Empatia”
Term 3	“Análise do Comportamento Aplicada”	“Applied Behavior Analysis”	“Análisis Aplicado de la Conducta”

^a^MeSH: Medical Subject Headings.

### Study Selection

The selected studies will focus on compassion and the area of concentration of applied behavior analysis, with a special focus on the compassion skills of the behavior analyst (Textbox 4).

All reviewers will undergo training on the application of inclusion or exclusion criteria before beginning the screening process to ensure consistency. The entire selection process will be piloted on a subset of 100 papers to refine the procedure before full implementation.

**This study selection process will follow these steps.**

**Duplicate removal:** After retrieving search results from all databases, 2 independent reviewers (DA and DS) will use Rayyan software (Qatar Computing Research Institute) [18] to identify and remove duplicate papers.

**Title and abstract screening:** The same 2 reviewers will independently screen titles and abstracts of the remaining study against the predefined inclusion and exclusion criteria. They will classify each paper as “include,” “exclude,” or “uncertain.”

**Full-text review:** For this study classified as “include” or “uncertain,” the full-texts will be retrieved and independently reviewed by the same 2 reviewers using the detailed eligibility criteria outlined in the protocol.

**Disagreement resolution:** Any disagreements at the title or abstract or full-text review stages will be resolved through discussion between the 2 reviewers. If consensus cannot be reached, a third reviewer (JA) will be consulted to make the final decision.

**Inter-rater reliability:** We will calculate the Cohen kappa coefficient to assess inter-rater reliability for both the title or abstract and full-text screening stages. A κ value of 0.8 or higher will be considered acceptable.

**Documentation:** Throughout the process, we will maintain a detailed log of all decisions, including reasons for exclusion at the full-text review stage. This information will be used to create the PRISMA (Preferred Reporting Items for Systematic Reviews and Meta-Analyses) flow diagram.

**Final verification:** As a quality control measure, a random sample of 10% of the included studies will be reviewed by a third reviewer (SC) to ensure consistency in application of the inclusion and exclusion criteria.

### Eligibility Criteria

In line with established methodological guidelines for scoping reviews, the analysis of study quality will not be considered as an eligibility criterion. This approach is consistent with the primary aim of scoping reviews, which is to map the existing literature on a topic rather than to assess the quality of individual studies [12].

The analysis of study quality will not be considered as an eligibility criterion, as scoping reviews do not include this assessment [13]. The initial reading of the title and abstracts will allow the exclusion of nonrelevant studies. Next, we will examine the full texts of studies that proved relevant or whose nature raised doubts.

For the calibration of eligibility criteria and to define search strategies among researchers, the title and abstract of 18 papers will be randomly analyzed by 2 researchers. There should be an 80% agreement on the inclusion and exclusion criteria of the papers. If the initial analysis does not achieve the 80% agreement threshold, the 2 researchers will discuss their discrepancies, refine their understanding of the inclusion and exclusion criteria, and repeat the process with a new set of 18 randomly selected papers. This iterative process will continue until the 80% agreement level is reached. Once achieved, this will establish the final set of eligibility criteria to be used for the full review process. This approach ensures a robust and consistent application of the eligibility criteria throughout the study selection phase.

### Data Extraction

The Rayyan software [18] will be used in the process of selecting evidence sources, where duplicates between databases and studies that do not address terms of the PCC acronym will be excluded.

Subsequently, the objectives and methods of the studies will be reviewed to verify if they meet the other eligibility criteria (Textbox 5).

These criteria will be applied during the full-text review phase to ensure that all included studies have a robust scientific basis in ABA.

Two reviewers (DA and DS) will independently extract data from each included study using a standardized data extraction form. The form will be piloted on a sample of 5 studies and refined as necessary before full implementation.

A comprehensive data extraction instrument has been developed to systematically collect relevant information from each included study, covering aspects such as study characteristics, ABA-specific information, compassion-related data, outcomes, themes, author conclusions, and quality assessment (Multimedia Appendix 1 for the full data extraction instrument).

Any disagreements in data extraction will be resolved through discussion between the 2 reviewers, with a third reviewer (JA) consulted if necessary. The extracted data will be compiled in a spreadsheet to facilitate thematic analysis and synthesis.

**To ensure that the selected studies have a strong scientific basis in applied behavior analysis, we will establish the following specific criteria.**

**Theoretical framework:** The study must explicitly state that it is grounded in behavioral principles or applied behavior analysis methodology.

**Methodology:** The study should employ research methods consistent with applied behavior analysis practices, such as single-subject designs, multiple baseline designs, or group designs that focus on behavioral outcomes.

**Terminology:** The study should use terminology consistent with applied behavior analysis, including but not limited to: operant conditioning, reinforcement, behavior modification, functional analysis, or behavioral intervention.

**Outcome measures:** The study should include behavioral measures as primary outcomes, such as frequency, duration, or intensity of target behaviors.

**Intervention techniques:** The study should describe intervention techniques that are consistent with applied behavior analysis principles, such as reinforcement schedules, prompting, shaping, or chaining.

**Professional standards:** The study should adhere to ethical guidelines and professional standards established by recognized applied behavior analysis organizations (eg, the behavior analyst certification board).

**Peer review:** The study must be published in a peer-reviewed journal that regularly publishes applied behavior analysis research or in a recognized behavioral science journal.

In addition, the studies should meet the following criteria:

- The studies will need to present in the title, abstract, or descriptors the concepts of applied behavior analysis and terms commonly used by researchers in the area, such as behavioral skills.
- The studies should present in the title, abstract, or descriptors the concepts of compassion or similar terms such as compassionate skills; however, they would need to be linked to applied behavior analysis.

### Data Analysis and Synthesis

We will use a thematic analysis approach to synthesize the extracted data (Textbox 6), following the 6-phase framework outlined by Braun and Clarke [19].

We will use both narrative synthesis and visual representations (eg, concept maps) to present our findings. The synthesis will aim to provide a comprehensive overview of the current state of knowledge regarding compassion skills in ABA, identify gaps in the literature, and suggest directions for future research.

**Data analysis process.**

**Familiarization with the data:** Two reviewers (DA and DS) will independently read and re-read the extracted data, noting initial ideas and potential codes.

**Generating initial codes:** Using MaxQDA software (VERBI Software), the reviewers will systematically code interesting features across the entire dataset. We will use a combination of deductive coding (based on our research questions) and inductive coding (allowing new themes to emerge from the data).

**Searching for themes:** Codes will be collated into potential themes, gathering all data relevant to each potential theme.

**Reviewing themes:** Themes will be checked in relation to the coded extracts (Level 1) and the entire dataset (Level 2), generating a thematic “map” of the analysis.

**Defining and naming themes:** Ongoing analysis to refine the specifics of each theme and the overall story the analysis tells, generating clear definitions and names for each theme.

**Producing the report:** Final analysis and selection of compelling extract examples, relating back to the research question and literature.

To ensure rigor and transparency in our analysis, we will:

- Use a codebook to maintain consistency in coding [20].
- Use investigator triangulation, with 2 reviewers independently coding and then comparing their analyses [21].
- Use member checking, where we will share our preliminary findings with a subset of included study authors for feedback [22].
- Maintain an audit trail of analytical decisions [23].

The types of information we will focus on during the analysis include:

- Conceptualizations of compassion within applied behavior analysis (ABA).
- Reported compassionate skills or behaviors in ABA practice.
- Impacts of compassionate approaches on ABA outcomes.
- Barriers and facilitators to implementing compassionate care in ABA.
- Emerging trends or gaps in the literature regarding compassion in ABA.

### Ethical Considerations

As this is a scoping review of existing literature and does not involve human participants, ethical approval is not required. However, ethical principles of research integrity will be followed, including accurate and complete reporting of methods and results.

## Results

The current status and planned timeline for the study are as follows:

- Protocol development: Completed in December 2024.
- Systematic literature search: Scheduled to commence in January 2025.
- Title and abstract screening: Planned for January-February 2025.
- Full-text review and data extraction: Planned for February-March 2025.
- Data analysis and synthesis: Expected to take place in April-May 2025.
- Final report writing: Scheduled for June-July 2025.
- Submission for peer review: Anticipated in August 2025.

As of this protocol publication, the selection of sources has not yet been initiated. Once the literature search is conducted, we will update this section with the number of papers retrieved and included in the review, using a PRISMA (Preferred Reporting Items for Systematic Reviews and Meta-Analyses) flow diagram to illustrate the selection process.

## Discussion

### Principal Findings

This scoping review aims to provide a comprehensive overview of compassion in the context of ABA. We expect to synthesize definitions and conceptualizations of compassion, map specific compassionate skills, analyze existing interventions and training programs, identify outcomes associated with compassionate practice, and highlight barriers and facilitators for implementing compassionate practices in ABA.

### Interpretation of Findings

The anticipated findings of this review have the potential to significantly impact ABA practice. By synthesizing existing evidence on compassionate skills in ABA, we expect to highlight the importance of integrating compassion into behavioral interventions. This could lead to a paradigm shift in ABA, promoting a more holistic and client-centered approach that balances behavioral techniques with empathetic care.

### Comparison With Existing Literature

While compassion has been extensively studied in other health care fields, its exploration in ABA is relatively new. Our review will build upon existing literature on compassion in health care, such as the work of Sinclair et al [8] on compassion in palliative care, and extend these concepts to the unique context of ABA. This review will also complement recent studies on the integration of “soft skills” in ABA practice [4], providing a more focused examination of compassion specifically.

### Strengths and Limitations

A significant strength of this study is its comprehensiveness, including a wide range of study types and not being limited to a specific geographical context. This will allow a global view of compassionate practices in ABA. However, a potential limitation is the possibility of missing relevant studies due to differences in terminology or inadequate indexing. To mitigate this, we will conduct a comprehensive search with varied terms and consult with experts in the field.

### Implications for Practice and Policy

The results of this scoping review have the potential to inform the development of compassion training programs for behavior analysts, improve clinical practices, and influence care policies in ABA. By identifying specific compassionate skills and existing training programs, we can provide a foundation for developing more comprehensive and effective compassion training in ABA education and professional development.

### Future Research Directions

This review is likely to identify gaps in the current understanding of compassion in ABA, providing direction for future empirical studies. Potential areas for future research may include developing and validating measures of compassion specific to ABA practice, conducting intervention studies to assess the impact of compassion training on client outcomes, and exploring the long-term effects of compassionate ABA practice on client well-being and therapeutic relationships.

### Conclusions

This scoping review represents a crucial step in understanding and promoting compassionate care within ABA. By synthesizing existing knowledge and identifying areas for future research, we aim to contribute to the evolution of ABA toward a more compassionate and holistic approach to behavioral intervention. This work underscores the importance of balancing technical proficiency with empathetic care in ABA practice, potentially leading to improved outcomes and experiences for clients and practitioners alike.

### Dissemination of Results

The results of this scoping review will be disseminated through publication in a peer-reviewed scientific journal. In addition, the findings will be presented at relevant ABA and psychology conferences. A summary of the results will be shared with professional ABA organizations to facilitate knowledge translation into practice.

## Appendix

Multimedia Appendix 1 Data extraction instrument for a scoping review on compassion in applied behavior analysis.

## Abbreviations

ABA: applied behavior analysis
ASD: autism spectrum disorder
MeSH: Medical Subject Headings
PCC: population, concept, and context
PRISMA: Preferred Reporting Items for Systematic Review and Meta-Analyses
PRISMA-ScR: Preferred Reporting Item for Systematic Review and Meta Analyses extension for Scoping Reviews
SCED: Single-Case Experimental Design

## Data Availability

This protocol has been registered on OSF Registries (osf.io/f3a6h) [24]. The datasets generated or analyzed during this study are available from the corresponding author on reasonable request.
