# Supplementary material for: Mapping the Evidence on Compassion Skills in Applied Behavior Analysis: Protocol for Scoping Review
Source: JMIR Res Protoc. 2025 Apr 24;14:e66399. doi: 10.2196/66399 (PMC12062760; doi:10.2196/66399)
Supplement: Multimedia Appendix 1 [file resprot_v14i1e66399_app1.docx]

Data Extraction Instrument for Scoping Review on Compassion in Applied Behavior Analysis

1. Study Characteristics:
   - Authors, year of publication, country of origin
   - Study design and methodology (e.g., qualitative, quantitative, mixed methods)
   - Sample size and participant characteristics (e.g., age, gender, diagnosis if applicable)
   - Study setting (e.g., clinical, educational, community, home-based)
   - Funding source and potential conflicts of interest
2. ABA-Specific Information:
   - Specific ABA techniques or interventions used
   - Target behaviors or skills addressed
   - Duration and intensity of ABA intervention
   - Qualifications and training of ABA practitioners involved
   - Theoretical framework or model of ABA used (if specified)
3. Compassion-Related Data:
   - Definition or conceptualization of compassion used in the study
   - Measures or assessments of compassion (if any)
   - Compassion-related skills or behaviors described
   - Training in compassionate care (if any)
   - Integration of compassion into ABA practice (methods and challenges)
4. Outcomes:
   - Primary and secondary outcomes reported
   - Measures used to assess outcomes
   - Key findings related to compassion in ABA practice
   - Statistical analyses used (if applicable)
   - Effect sizes or other measures of impact (if reported)
5. Themes and Concepts:
   - Main themes or concepts related to compassion in ABA
   - Barriers or facilitators to implementing compassionate care in ABA
   - Reported impacts of compassionate approaches on ABA outcomes
   - Ethical considerations related to compassionate care in ABA
6. Author Conclusions:
   - Key conclusions drawn by the authors
   - Implications for practice or future research suggested
   - Recommendations for policy or guideline development
7. Quality Assessment (for descriptive purposes only):
   - Study limitations reported by authors
   - Potential sources of bias identified
   - Generalizability of findings
   - Adherence to reporting guidelines (e.g., CONSORT, STROBE, COREQ)
8. Reviewer Notes:
   - Any additional observations or interpretations by the reviewer
   - Questions or uncertainties to be discussed with the research team
   - Potential links or contradictions with other included studies
